# Neonatal Clinical Pharmacology

**DOI:** 10.3390/children11091102

**Published:** 2024-09-09

**Authors:** Karel Allegaert, Nadir Yalcin, Robert B. Flint, Sinno H. P. Simons

**Affiliations:** 1 Department of Pharmaceutical and Pharmacological Sciences, KU Leuven, 3000 Leuven, Belgium; 2 Department of Development and Regeneration, KU Leuven, 3000 Leuven, Belgium; 3 Department of Hospital Pharmacy, Erasmus Medical Center, 3015 GD Rotterdam, The Netherlands; r.flint@erasmusmc.nl; 4 Department of Clinical Pharmacy, Faculty of Pharmacy, Hacettepe University, Ankara 06100, Türkiye; nadir.yalcin@hacettepe.edu.tr; 5 Rotterdam Clinical Pharmacometrics Group, Erasmus University Medical Centre, 3015 GD Rotterdam, The Netherlands; 6 Department of Paediatric and Neonatal Intensive Care, Division of Neonatology, Erasmus University Medical Centre-Sophia Children’s Hospital, 3015 GD Rotterdam, The Netherlands; s.simons@erasmusmc.nl

Similar to other populations, care of neonates strongly relies on robust guidance on neonatal pharmacotherapy, covering an age-appropriate drug formulation, an individualized dose, and information on its efficacy and safety for a specific indication in this population, preferably weighted to alternative approaches [1]. In contrast to other populations, the majority of our current drug prescription practices in neonates are still off-label or unlicensed. Hopefully most prescriptions are guided by guidelines and evidence-based neonatal drug formularies, so that off-label does not necessarily reflect off-knowledge practices, as these doses can still be supported by sufficient and reliable information [2,3]. In a recently published paper in this journal, a global scope has been reported on the available neonatal drug formularies and their workflow and background. Eight different neonatal drug formularies were identified worldwide (Europe, USA, Australia-New Zealand, Middle East) [2]. Six provided their response to the questionnaire and were compared for structure and content. It turned out that each drug formulary has its own workflow, monograph template and style, and update routine. Focus on certain aspects of drug interactions also varies, as well as the type of initiative and funding. Consequently, health care providers working in the neonatal field should be aware of the various drug formularies available and their differences in characteristics and content to use them properly for the benefit of their patients [2].

The Neodose project hereby suggests a 3-step approach to further develop neonatal drug formularies, starting with priority (frequency of prescription, advice already available, prescribers’ preferences or perceived needs), followed by establishment of a consensus-based (local practices and experiences) dosing recommendation, and a subsequent best-evidence dosing (literature review on evidence) recommendation. This workflow turned out to be feasible and relevant, as illustrated for 37 dosing recommendations, representing 19 indications in preterm (*n* = 22) or term (*n* = 15) neonates, respectively [3].

Related to the global scope on neonatal drug formularies, two additional comments (1,2) were published in the current Special Issue. These comments mention two additional neonatal drug formularies. One (Swiss database, www.swisspeddose.ch/database, accessed on 14 August 2024) was identified at the initial screening [2] and described their workflow, applying the same approach as described in the Global Scope paper (1) [2]. We were unaware of a second formulary (Ibero-American Society of Neonatology (SIBEN), NEOFARMA-SIBEN), but were able to add this formulary to the list (2).

As already mentioned, any drug formulary should be informed by clinical studies in its broader understanding, including real-world and observational data, to be able to come up with evidence-based dosing recommendations, as well as safety and efficacy aspects. Three papers have been published in this Special Issue, reporting on a review on pharmacological management of neonatal hypotension (3) and a safety study exploring the association of inotrope administration, fentanyl use, and intraventricular hemorrhage in very low gestational age infants (4). Finally, the suitability of a gentamicin dosing strategy in neonates, driven by a population pharmacokinetic approach with truncated sampling duration was also published (5).

The very detailed update on neonatal hypotension management hereby reminds us again on the ‘common’ issues in neonatal pharmacology and management, namely the knowledge gaps (reference values on normal blood pressure and on clinically relevant hypotension values, the add-on values of new techniques to assess hemodynamics) and management gaps (drug selection, dose selection) (3). In the retrospective observational study, fentanyl administration, severe intracranial hemorrhage and lower gestational age were risk factors strongly associated with the clinical perceived need for inotrope treatment in very low gestational age infants, while cause-consequence explorations and causality assessment are obviously not possible in a retrospective study design (4). The paper on gentamicin dosing strategy is a very nice example of quality improvement ‘in the field’, capturing the specific characteristics of a given cohort, to subsequently explore the performance of a dosing (in this case, 5 mg/kg/24 h) regimen (5). This information can subsequently be incorporated to develop or adapt neonatal drug formularies, and our clinical practices.

As a closing reflection, care providers should realize that pharmacotherapy is a very powerful intervention to improve outcomes, and this is also true in neonates. The prescription of a given drug should result in a safe and effective intervention to treat or prevent a specific disease or risk in an individual patient or population while avoiding disproportional side-effects. Clinical pharmacology supports these aims in predicting drug-related (side) effects driven by pharmacokinetics (PK) and pharmacodynamics (PD). The dynamic changes related to maturation and growth in newborns result in a unique setting with extensive variability. Moreover, optimal and valid PD targets in (preterm) neonates often have not yet been established or are hampered to readily use for treatment evaluation. Non-maturational changes (such as disease characteristics, drug–drug interactions, pharmacogenetics, and lactation-related exposure) further add to this variability. The most effective way to improve this is to share practices, compare outcomes, and conduct the relevant studies in this population. We strongly believe that newly emerging research tools like machine learning and real-world data analysis will become instrumental in taking the additional steps needed to improve neonatal pharmacotherapy.
